# Combined treatment of All-trans retinoic acid with Tamoxifen suppresses ovarian cancer

**DOI:** 10.1007/s00280-024-04671-7

**Published:** 2024-05-07

**Authors:** Rui Xu, Xiaowen Yang, Bin Tang, Yifan Mao, Feiyun Jiang

**Affiliations:** 1 Department of Gynecology, East China Normal University Wuhu Affiliated Hospital, The Second People’s Hospital of Wuhu City, No.259, Middle Jiuhua Road, Jinghu District, Wuhu, 241000 China; 2 Department of Electrocardiogram, East China Normal University Wuhu Affiliated Hospital, The Second People’s Hospital of Wuhu City, No.259, Middle Jiuhua Road, Jinghu District, Wuhu, 241000 China

**Keywords:** Ovarian cancer, Tamoxifen, ATRA, Combination therapy

## Abstract

**Background:**

Ovarian cancer is a malignant tumor of the female reproductive system, and its mortality rate is as high as 70%. Estrogen receptor α (ERα)-positive ovarian cancer accounted for most of all ovarian cancer patients. ERα can promote the growth and proliferation of tumors.

**Methods:**

The combined effect of All-trans retinoic acid (ATRA) and tamoxifen was obtained by the combination screening of tamoxifen and compound library by MTS. In addition, colony formation assay, flow cytometry analysis, immunofluorescence staining, quantitative real-time polymerase chain reaction (PCR), western blot, and tumor xenotransplantation models were used to further evaluate the efficacy of tamoxifen and ATRA in vitro and in vivo for ER-α-positive ovarian cancer.

**Results:**

In our study, we found that All-trans retinoic acid (ATRA) can cooperate with tamoxifen to cause cell cycle arrest and apoptosis and inhibit ERα-positive ovarian cancer in vivo and in vitro. Further exploration of the mechanism found that ATRA can Inhibit genes related to the ERα signaling pathway, enhance the sensitivity of ERα-positive ovarian cancer cells to tamoxifen, and ascertain the effectiveness of tamoxifen and ATRA as treatments for ovarian cancer with an ERα-positive status.

**Conclusion:**

Combination of ATRA and tamoxifen is a new way for the treatment of ERα-positive ovarian cancer.

## Introduction

In the reproductive system of women, ovarian cancer is one of the most common malignant tumors. Ovarian cancer incidence and mortality rates vary globally and are influenced by many factors. In general, the incidence rate is higher in developed countries and relatively lower in developing countries. The World Health Organization (WHO) estimates that around 250,000 women across the globe receive a diagnosis of ovarian cancer annually. The five-year survival rate is only 25% [1] and most patients have advanced to the advanced stage when they are diagnosed with ovarian cancer [2]. Ovarian cancer is often divided into different subtypes based on histological features and molecular markers. One of the subtypes is ERα-positive ovarian cancer. Patients with ERα-positive ovarian cancer account for more than 60% of ovarian cancer patients [3], and there is no complete understanding of its pathogenesis, but it is believed to be associated with hormone signaling and estrogen. Multiple studies have investigated the expression of ERα in epithelial ovarian cancer, but the largest is the study reported by Sieh et al. in 2013. This investigated 2933 women and identified ERα positivity in 81% of HGSOCs, 88% of LGSOCs and 77% of endometrioid ovarian carcinomas [4, 5]. Activation of estrogen receptors by estrogen can promote cell proliferation and survival in ERα-positive cancer cells [6]. Therefore, blocking or inhibiting estrogen signaling may be a therapeutic approach for this subtype of ovarian cancer.

Endocrine therapy for ERα-positive ovaries shares some similarities with ERα-positive breast cancer in that endocrine therapy uses drugs that block estrogen receptors or lower estrogen levels to slow or stop the growth of ERα-positive cancer cells. Tamoxifen is a well-known selective estrogen receptor modulator (SERM) for breast cancer, whose therapeutic mechanism is largely based on its ability to interact with estrogen receptors in the body [7]. Tamoxifen binds to estrogen receptors present in breast tissue and other target organs, especially estrogen receptors alpha (ERα) and beta (ERβ), once bound to estrogen receptors, tamoxifen Acts as an antagonist, blocking the ability of the receptor to be activated by estrogen, which results in a decrease in estrogen-mediated cell growth signaling [8]. By blocking estrogen receptors, tamoxifen deprives estrogen-sensitive cancer cells of the estrogen they need to grow and proliferate. In addition to blocking estrogen receptor activation, tamoxifen may induce apoptosis in some breast cancer cells, causing them to be destroyed. A common use of tamoxifen is as an adjuvant therapy in the treatment of metastatic breast cancer, which reduces the risk of cancer recurrence and improves overall survival [9]. Tamoxifen has been studied in ovarian cancer, a study reported 30 patients with persistent or recurrent epithelial ovarian Treated with tamoxifen after chemotherapy with Plantinum. Two complete remissions (duration 41 and 12 months, respectively) were recorded (6.6%), while 10 patients (33.3%) had stable disease for a mean duration of 11.5 months. Tamoxifen is a reasonable treatment option for patients with persistent or recurrent ovarian cancer [10].

All-trans retinoic acid (ATRA)is a vitamin A acid analog, also known as retinoic acid. ATRA mainly regulates gene expression by binding retinoic acid receptors (RARs), thereby affecting cell differentiation, proliferation, and apoptosis [11]. In terms of tumor treatment, ATRA has made a remarkable breakthrough in the treatment of acute promyelocytic leukemia [12] (APL). In addition to APL, ATRA has also been studied and applied in the treatment of other types of tumors. For example, ATRA has promising potential as a novel therapy against serous ovarian cancer [13] and as a potential anticancer drug in the sub-group of ovarian carcinomas in which the TERT promoter is hypomethylated [14] and inhibits HGSOC cell growth by inducing Pin1 degradation [15]. Studies have shown that ATRA may inhibit tumor growth and spread through different pathways, such as regulating cell cycle, promoting apoptosis, and inhibiting angiogenesis [16]. However, the efficacy of ATRA in these tumor types still needs further research and validation. ATRA is often used in combination with chemotherapy drugs to improve efficacy. In acute myeloid leukemia AML, the combination of ATRA and ATO enhances the differentiation and cell death induction of APL cells [17]. In colorectal cancer (CRC) It is a life-threatening malignant tumor, it has been found to be resistant to 5-fluorouracil (5-FU). ATRA can enhance the inhibitory effect of 5-FU on colorectal cancer cells and promote cell apoptosis [18]. There has also been research showing that ATRA inhibits ERα protein expression in breast cancer cells, and breast cancers resistant to tamoxifen may be inhibited by the drug combination with tamoxifen [19], but the combination effect and mechanism in ovarian cancer are still unclear.

Through the screening of the drug library, we determined that the combined use of ATRA and tamoxifen can synergistically inhibit the proliferation of ERα-positive ovarian cancer, and explored the mechanism by which the combined drug promotes the death of ERα-positive ovarian cancer, and clarified the role of ATRA and tamoxifen. Combination of ATRA and tamoxifen is a new way for the treatment of ERα-positive ovarian cancer.

## Materials and methods

### Cell lines and cell culture

American Typical Cell Culture (ATCC) was the source of ovarian cancer cells SKOV3, PEO-1, and CAOV3. As a culture medium, 10% fetal bovine serum (FBS) and 1% penicillin/streptomycin were added to RPMI-1640 medium for SKOV3 cells. In addition to 10% fetal bovine serum, 10% glucose, and 1% penicillin/streptomycin, cell culture medium was used for PEO-1 and CAOV3 cells. 37 °C, 5%CO2, and 95% humidity were the culture environments for these cell lines. The source of ATRA (all-trans retinoic acid) and TAM (tamoxifen) was MedChemExpress.

### Cell proliferation assay

Cell proliferation assays were performed at 37˚C. SKOV3, PEO-1 and CAOV3 cells were inoculated into 96‑well plates with a density of 5 × 10^3^ cells per well. The cells were incubated overnight in a 37˚C, 5%CO2 incubator, observed to assess adhesion and respectively treated with ATRA and TAM after cell adhesion, for 72 h. A total of 20 µl MTS was added to each well away from light and incubated in a 37˚C incubator for 20‑50 min. The optical density (OD) of each well was measured at 490 nm using an Spectra Max 190 enzyme spectrometer (Molecular Devices LLC), and the OD of all samples were recorded when the OD of the control reached 0.8‑1.2. MTS viability assays measures cell proliferation by measuring cell metabolism.

### Colony formation rates

Five hundred and three cells per well were seeded in 6-well cell culture plates for SKOV3 and PEO-1 cells, adding 10% FBS to medium and incubating overnight in triplicate. In the experimental group, fresh medium containing drug was added to maintain a certain drug concentration, and the control group was also treated with DMSO at the same volume. A visible colony was observed by naked eye after one week at 37 °C. After cloning had occurred, cells were fixed with paraformaldehyde for about 25 min, in the subsequent step, the cells were washed with PBS and stained for 15 to 20 min with 2% crystal violet solution to color them. Finally, an excess of staining solution was washed off the surface of the cells and dried by air. A count of cell colonies was performed in the Wells, rate of colony formation (%)/rate of colony formation (control) (%) was used to calculate colony formation rates.

### Flow cytometry

SKOV3 and PHO-1 cells in the experimental group were seeded in medium containing a certain concentration of ATRA and TAM, and In the control group, DMSO was added at the same volume. A 48-h treatment period was followed by the collection of supernatants and digested cell suspensions. At room temperature in the dark, whole cells in the binding buffer suspension were stained with 1 µL RNA enzyme (Sigma, USA), 2 μL annexin V–FITC (BD, USA), and 2 μL propidium iodide (PI) (Sigma, USA) for 15 min. Cells unstained and those stained once served as controls. Flow cytometry cups were used to examine these samples, and FACS Calibur (BD) was used to analyze stained cell. FlowJo software (v10) was used to analyze the data.

### Western blot analysis

10-cm dishes were used to seed and culture SKOV3 and PEO-1 cells. Cells were collected after 48 h of drug treatment. RIPA lysis buffer (Sigma) was used to extract proteins, and BCA assay was used to determine protein concentrations. SDS-PAGE (Sanguang Bioengineering Technology Services, Shanghai, China) was performed by means of the operating instructions on the Cell Signaling Technologies website. Western blotting was performed using antibodies against BCL2 (T40056F, ABWAYS), BCL-XL (2764S, Cell Signaling Technology), CyclinB1 (12231S, Cell Signaling Technology), CyclinE1 (4129 T, Cell Signaling Technology), c-MYC (CY5150, ABWAYS), γ-H2AX (9718S, Cell Signaling Technology), GAPDH (AB0036, ABWAYS). Anti-rabbit 800 and mouse 800 (LI-COR Biosciences). Imaging of the membrane was carried out using an Odyssey infrared imaging system (LI-COR Biosciences), and measurements were made using Image Studio Lite software.

### Immunofluorescence

Eighty-three thousand cells were seeded into each well of a 24-well plate, and a cover slip was placed on top. Different concentrations of ATRA and Tamoxifen were used to treat the cells. After treatment, incubation was started at 37 °C, 5%CO2 and 95% humidity for 48 h. 0.2%Triton (Sangon, China) in 1 × PBS was used to fix the cells and permeabilized for 30 min. 1%BSA (Sangon) in 0.2%Triton/PBS was used to incubate the cells for 30 min. Primary rabbit anti-γH2AX antibody (1:400) was then used to incubate the cells overnight at 4° C. 0.2%triton/PBS was then used to wash the cells three times for 3 min each, and a second anti-rabbit 800 antibody was used to incubate with the cells for 1 h in the dark. DAPI (D9542, Sigma) was used to counterstain the nuclei for 5 min, and for three 5 min-washings, 0.2% triton/PBS was used. An Olympus inverted fluorescence microscope was used to capture images.

### Quantitative real-time PCR (RT-qPCR)

ATRA or TAM was used to treat SKOV3, PEO-1, and CAOV3 cells for 24 h, and isolation of total RNA was performed using TRIzol reagent. RNA was extracted, and a reverse transcription reaction using Prime Script RT kit was used to produce cDNA from the extracted RNA. Reverse transcription of cDNA served as the template for the RT-qPCR reaction, and this reaction was detected using SYBR Green on the QuantStudio® 3 real-time PCR system. The internal control was GAPDH. The conditions for the reaction were as follows: In the first step, 25 °C is kept for 5 min, in the second step, 42 °C is kept for 30 min, in the third step, 85 °C is kept for 5 min, and in the final step, 16 °C is kept. In the PCR profile, the first reaction took place for 2 min at 95 °C, followed by 40 cycles at 95 °C for 10 SEC and 60 °C for 30 SEC. GraphPad Prism was used to analyze the data, and the 2-ΔΔCT method was used to calculate relative gene expression.

### Xenograft tumor growth

1 × 10^7^ SKOV3 cells were injected subcutaneously with Matrigel at a ratio of 1:1 in 6 ~ 8 week old female nude mice. When the average tumor volume reached 100 mm^3^, the mice were randomly grouped and then administered by intraperitoneal injection for 36 d. The body weights and tumor sizes of the rats were measured. Tumour volume was calculated by the formula: tumor volume = length × width2 × 0.52. Mice were executed after drug administration, and the tumors and major organs were dissected and collected for subsequent experiments.

### Statistical analysis

The mean ± SD is presented as the result. All experiments presented in this article were performed at least three times to make the results more reliable, except for the animal experiments. In order to determine whether the differences between the two groups were statistically significant, we used the Student t test. Statisticians used GraphPad Prism 8.0 to analyze the data. In *P < 0.05, P < 0.01, P < 0.001 and ******P < 0.0001 level to determine the mean significant difference.

## Results

### Screening for ATRA that has potential synergistic activity with tamoxifen to inhibit ovarian cancer cells

**Fig. 1:**
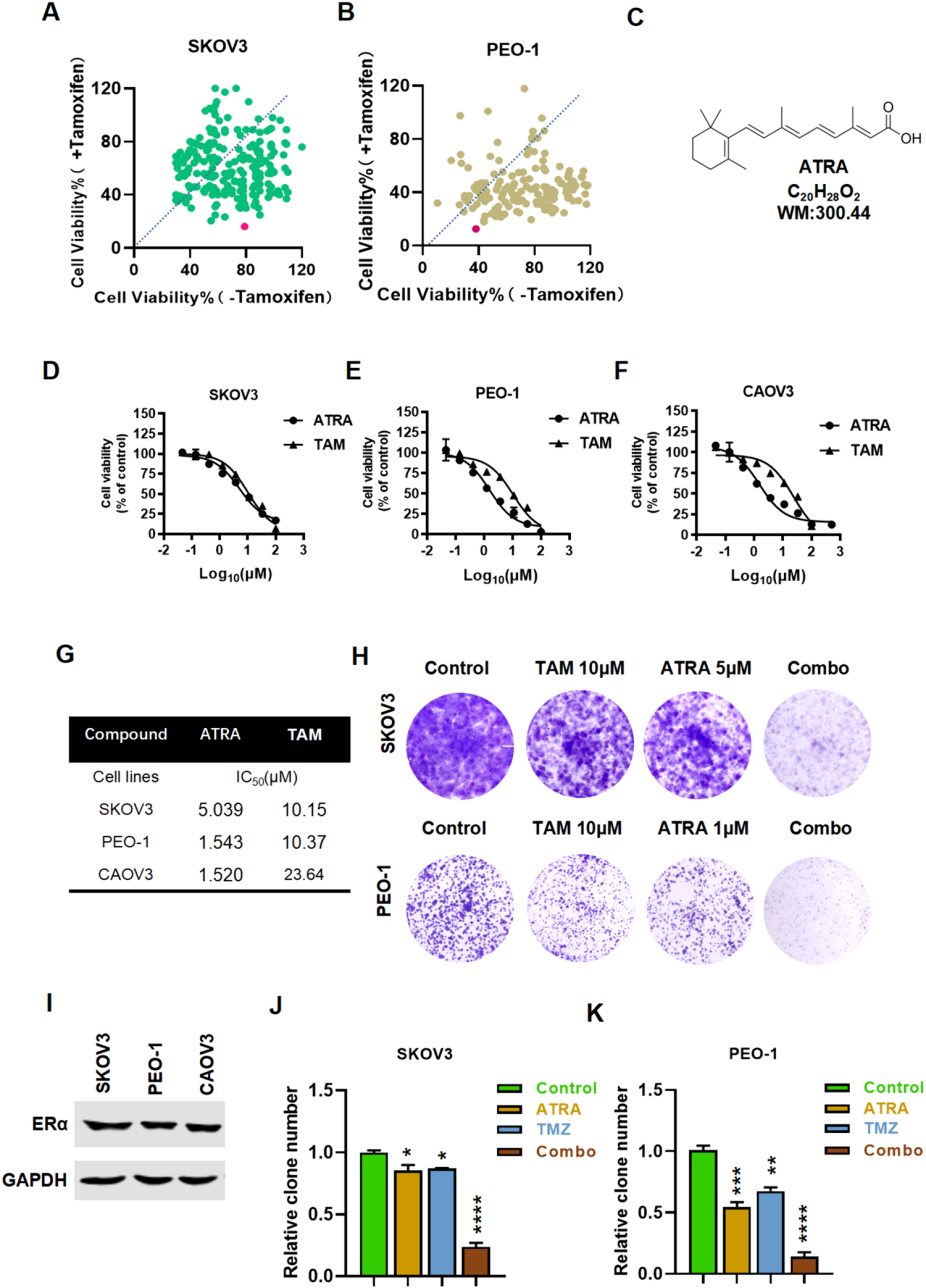
Screening of candidate compounds and the inhibitory activity of ATRA and Tamoxifen on the proliferation of ovarian cancer cells SKOV3 and PEO-1 cell line screening results, red dots marked ATRA. The orange dotted line shows a slope of 1, and points below the dotted line represent lower survival rates in the combination group than in the single-drug groups (**A** and **B**). Chemical structure of compound ATRA (**C**). MTS viability assays were performed on ovarian cancer and control cells treated for 72 h with ATRA or TAM. IC50 values were calculated using GraphPad (**D-G**). ATRA and Tamoxifen synergistically inhibit ovarian cancer cell colony proliferation, and Tamoxifen was combined with ATRA or administered alone to SKOV3 and PEO-1 cells to assess the effects on clonal proliferation. Representative images of SKOV3 and PEO-1 colonies are shown (**H**). The expression of ERα was detected in SKOV3、PEO-1 and CAOV3 (**I**). **J-K** is the statistical histogram of the colony formation rate in Figure H. Three times the data were collected and the mean and standard deviation were calculated. Unpaired t-test: ***P<0.001, ****P<0.0001

We performed a preliminary screening of 500 compounds in the existing FDA/CFDA compound library in our laboratory. In SKOV3 cells, there were 96 candidate compounds with a combined/ single-agent group survival ratio of less than 1, compared with 170 on PEO-1 cells. ATRA was one of the compounds with the smallest ratio, and its survival rate of the single-agent and combination groups were (79.89%, 18.61%) and (39.28%, 11.58%) in SKOV3 and PEO-1 (Fig. 1A, B). As shown in Fig. 1C, ATRA has a chemical structure. Therefore, we used ATRA as candidate for the next experimental validation. IC_50_ of ATRA and TAM were determined in three ovarian cancer cell lines (Fig. 1D-G). The results of cloning experiments showed that ATRA combination of Tamoxifen could inhibit the colony formation of SKOV3 and PEO-1 cells, and there was a significant difference between the single-agent and the combined inhibitory effect. The combination group of ATRA 5 μM in SKOV3 and ATRA 1 μM in PEO-1 reduced the average colony formation rate to 18.04% and 9.33% (Fig. 1H-J).

### The ATRA combined with tamoxifen showed significant synergy in ovarian cancer cells

**Fig. 2:**
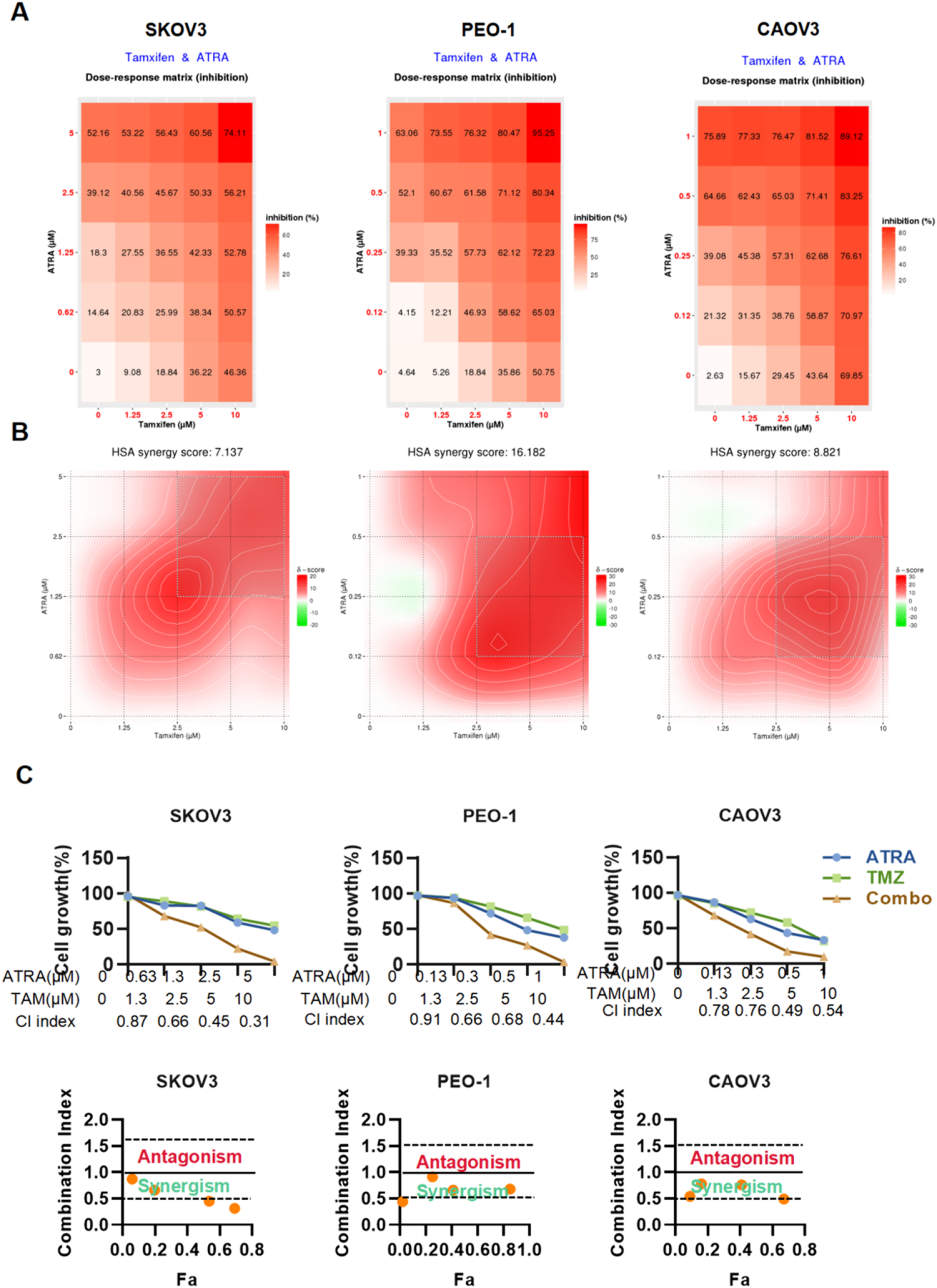
Effect of ATRA in combination with Tamoxifen on ovarian cancer cell growth. Changes in inhibition rates of SKOV3, PEO-1, and CAOV3 cell lines treated with ATRA combined with tamoxifen (**A**), Combined matrices show changes in the proliferation of SKOV3, PEO-1, and CAOV3 cell lines after treatment with ATRA in combination with tamoxifen (**B**). Synergy scores were calculated using Synergyfinder software. The HSA (highest single agent) algorithm was used to calculate the collaborative score, and the color key was used to represent the score in the heatmap. Proliferation plots in SKOV3, PEO-1, and CAOV3 cell lines after treatment with ATRA and tamoxifen, respectively (**C**), Shown below the columns are plots of CI versus inhibition rate, the fraction affected, Fa, for different conditions. Points with CI below 1 fall in the green “synergistic” region, and points with CI above 1 fall in the red “antagonistic” region. The mean is expressed as the sum of the standard deviations. (n=4 per group)

Next, to examine the effect of combined use of ATRA and tamoxifen, we examined the changes in the inhibition rate of three cell lines SKOV3, PEO-1 and CAOV3 after combined use. Cells were inhibited more efficiently as drug concentrations increased (Fig. 2A). HSA (highest single agent) algorithm was used to calculate synergy scores, and color keys indicate the scores in heatmaps. The results showed a synergistic effect of ATRA and Tamoxifen (Fig. 2B). In SKOV3, PEO-1 and CAOV3 cells treated with different concentrations of ATRA and Tamoxifen, the proliferation of cells was inhibited, and with the increase of drug concentration, the inhibitory effect on cell proliferation was more obvious (Fig. 2C). Moreover, the cells treated with ATRA combined with Tamoxifen had a more obvious inhibitory effect than those treated with ATRA and Tamoxifen alone, suggesting that Tamoxifen and ATRA work synergistically. The mean CI values of ATRA combination of Tamoxifen in SKOV3, PEO-1 and CAOV3 were 0.56, 0.77 and 0.68 (Fig. 2C). The values were all less than 1, and inhibition of ovarian cancer cells by ATRA and Tamoxifen is universal, indicating their synergistic effects.

### The ATRA combined with tamoxifen were synergistic in increasing apoptosis of SKOV3 and PEO-1 cells.

**Fig. 3:**
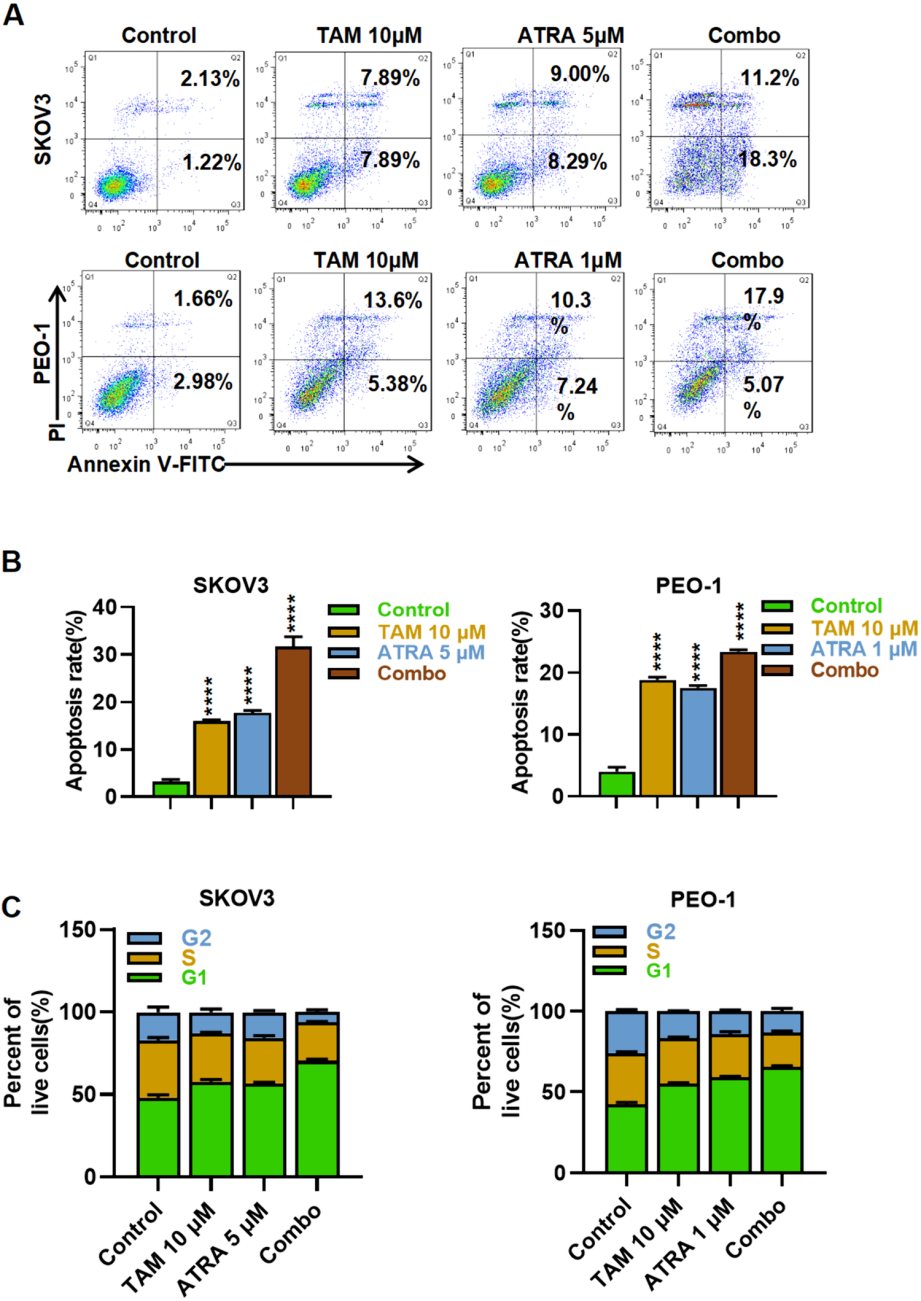
The effect of ATRA in combination with Tamoxifen on apoptosis and cell cycle arrest of ovarian cancer cells. In 48-hour treatments with ATRA and TAM alone and in combination, SKOV3 and PEO-1 cells exhibit apoptosis (**A**). Data statistics of SKOV3 and PEO-1 apoptosis (**B**). Effects of different concentrations of ATRA in combination with Tamoxifen on G1, S and G2 cell cycle proportions of SKOV3 and PEO-1 cells, Data Statistics of SKOV3 and PEO-1 cell cycle (**C**). Data are expressed as mean ± SD, *P < 0.05, ***P < 0.001, ****P < 0.0001, n.s. analysis of variance by single factor and multiple comparisons did not reveal any significant results

To further demonstrate the synergistic effects of ATRA and tamoxifen, after treatment with either ATRA alone or tamoxifen alone, SKOV3 and PEO-1 cells were measured for apoptosis compared with combined treatment. Treatment with ATRA (5 µM) plus tamoxifen (10 µM) resulted in a total apoptotic rate of 29.5% for SKOV3 cells, while that of tamoxifen alone was 15.78%, and that of ATRA alone was17.29%. Accordingly, the combination group's apoptosis rate was approximately twofold higher than the monotherapy group. In PEO-1, the total apoptosis rate was 22.97% when ATRA (1 µM) was combined with tamoxifen 10 µM), while the apoptosis rate was 18.98% when tamoxifen was used alone and 17.54% when ATRA was used alone (Fig. 3A, B). ATRA combined with tamoxifen treatment prolonged the G1 cell cycle proportions and shortened the S and G2 cell cycle proportions of SKOV3 and PEO-1 cells (Fig. 3C). These results indicated that the apoptosis rate of SKOV3 and PEO-1 cells was increased with the combination of ATRA and tamoxifen, which was consistent with the in vitro cell culture results.

### The ATRA combined with tamoxifen synergistically increased double-stranded DNA damage

**Fig. 4:**
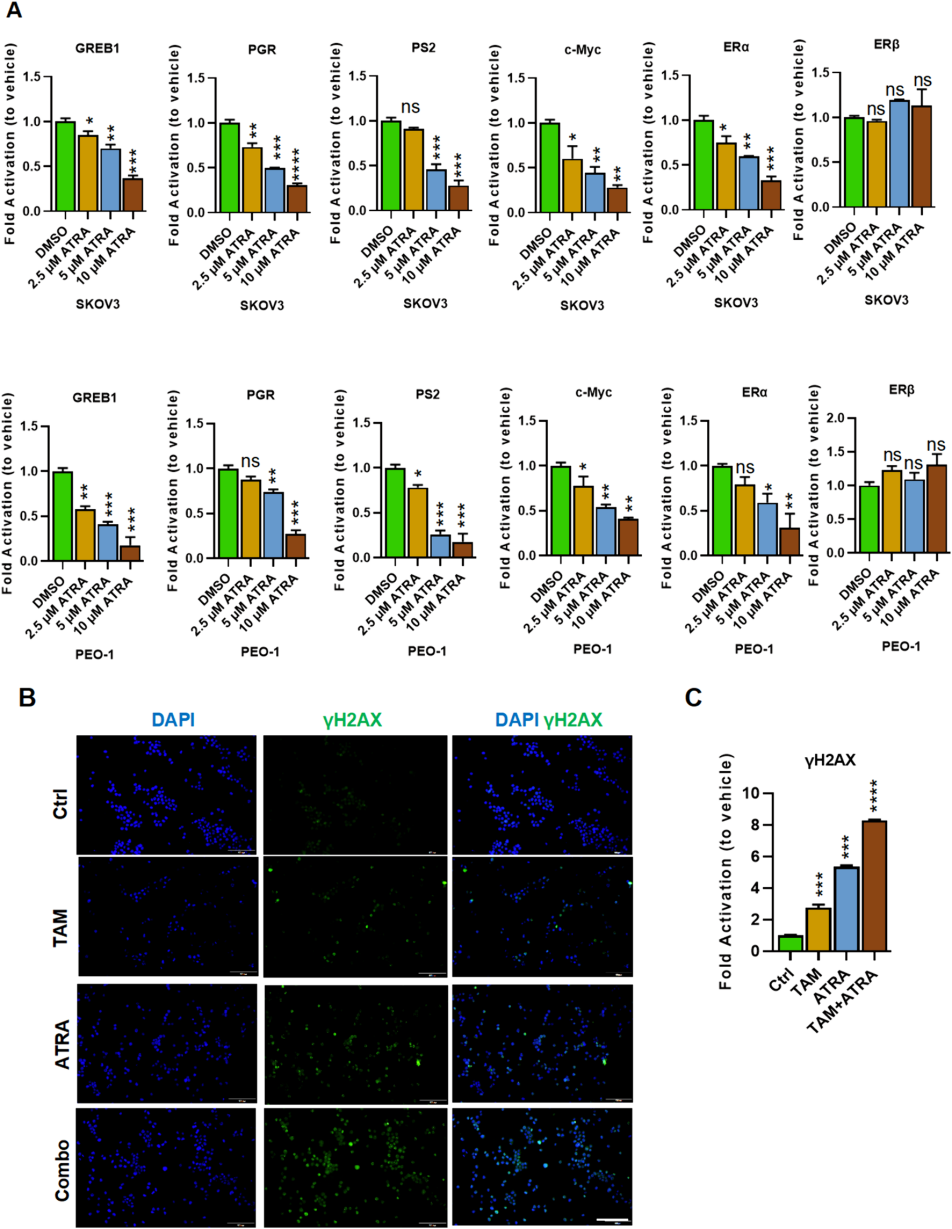
Effect of ATRA on expression of ERα-related genes in SKOV3 and PEO-1. Comparison of ATRA treatment with control to determine the fold change in inhibition. Figure for each gene (**A**). Cells treated with ATRA combined with TAM were subjected to immunofluorescence staining for γ-H2AX (**B**). Scale bars are 50 μm. Three times the data were collected and the mean and standard deviation were calculated. Unpaired t-test, ns, no significant difference; *P<0.05, **P<0.01, ***P<0.001, ****P<0.0001. γH2AX integrated density were calculated by Image J software (**C**)

**Fig. 5:**
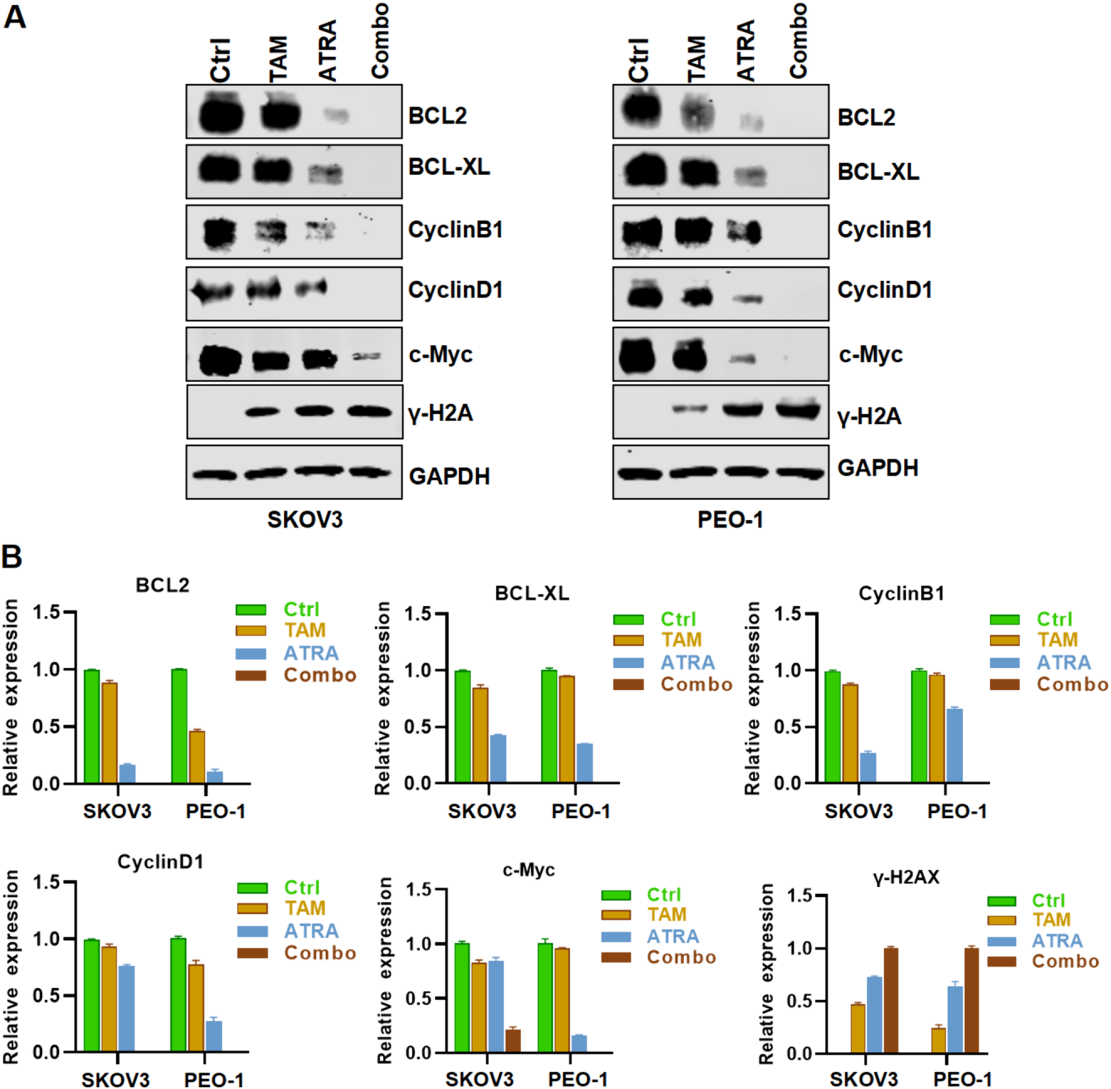
Protein expression of apoptosis and DNA damage in the presence of ATRA and TAM. SKOV3 and PEO-1 cells treated with ATRA and TAM 10 μM alone or in combination. ATRA 5μM in SKOV3 and ATRA 1μM in PEO-1. An analysis of the expression of BCL2, BCL-XL, CyclinB1, CyclinD1, c-Myc, and γ-H2AX in SKOV3 and PEO-1 cells treated with ATRA and TAM alone or in combination was conducted using Western blots (**A**). Densitometry analysis of these protein levels normalized to GAPDH (**B**)

As described above, the combination significantly enhanced the level of apoptosis. In order to further verify the pro-apoptotic effect of ATRA combined with Tamoxifen on ovarian cancer cells at the molecular level, the effects of different ATRA concentrations on genes related to ERα expression, such as PGR, PS2 and c-Myc were counted. Expression of genes associated with the ERα was significantly inhibited with the increase of drug concentration. We also examined the expression of ERα and ERβ, and found that ATRA treatment inhibited the expression of Erα in a concentration-gradient manner, but the expression of ERβ was not significantly changed.(Fig. 4 A). There are many variants of histone H2A, but H2AX has the greatest conservation, moving to the DNA double-strand damage core and phosphorylates its Ser139 residues, phosphorylation of H2AX termed γH2AX, an indicator of DNA double-strand breaks that is widely recognized. Moreover, this phosphorylation is reversible, and dephosphorylation occurs after DNA double-strand break repair. As shown in Fig. 5A, the significant increase of γH2AX protein expression in the combination groups, indicating that the combination of the two drugs indeed increased unrepaired DNA double-strand damage in SKOV3 and PEO-1, which coincides with the detection of apoptosis.

Phosphorylation of H2AX spreads from near to far from DNA double-strand breaks, and the resulting γH2AX foci is predicted to contain possible 2 Mb regions. Many DNA repair and/or cell cycle checkpoint protein components will accumulate on growing H2AX foci, which may help open chromatin structure and form a platform for accumulating DNA damage response and repair factors.

Thus, we performed the immunofluorescence experiments, according to the results, the combination group had a higher percentage of H2AX foci than either group of ATRA or Tamoxifen alone (Fig. 4B-C), indicating that the combination of ATRA and Tamoxifen ultimately played the effect of enhancing DNA double-strand damage.

### ATRA combined with tamoxifen inhibited the expression of apoptotic and cycle-related proteins.

An analysis of the expression of proteins related to apoptosis and the cell cycle detected by Western Blot showed that the expression of cell cycle-promoting proteins BCL2, BCL-XL, CyclinD1, CyclinE1 and c-Myc in the cells treated with ATRA and tamoxifen was significantly decreased, while the expression of γH2AX was significantly increased (Fig. 5A, B). Such results further confirmed the pro-apoptotic effect of ATRA combined with tamoxifen on ovarian cells at the protein level.

### ATRA combined with tamoxifen suppresses growth of ovarian cancer in vivo

**Fig. 6:**
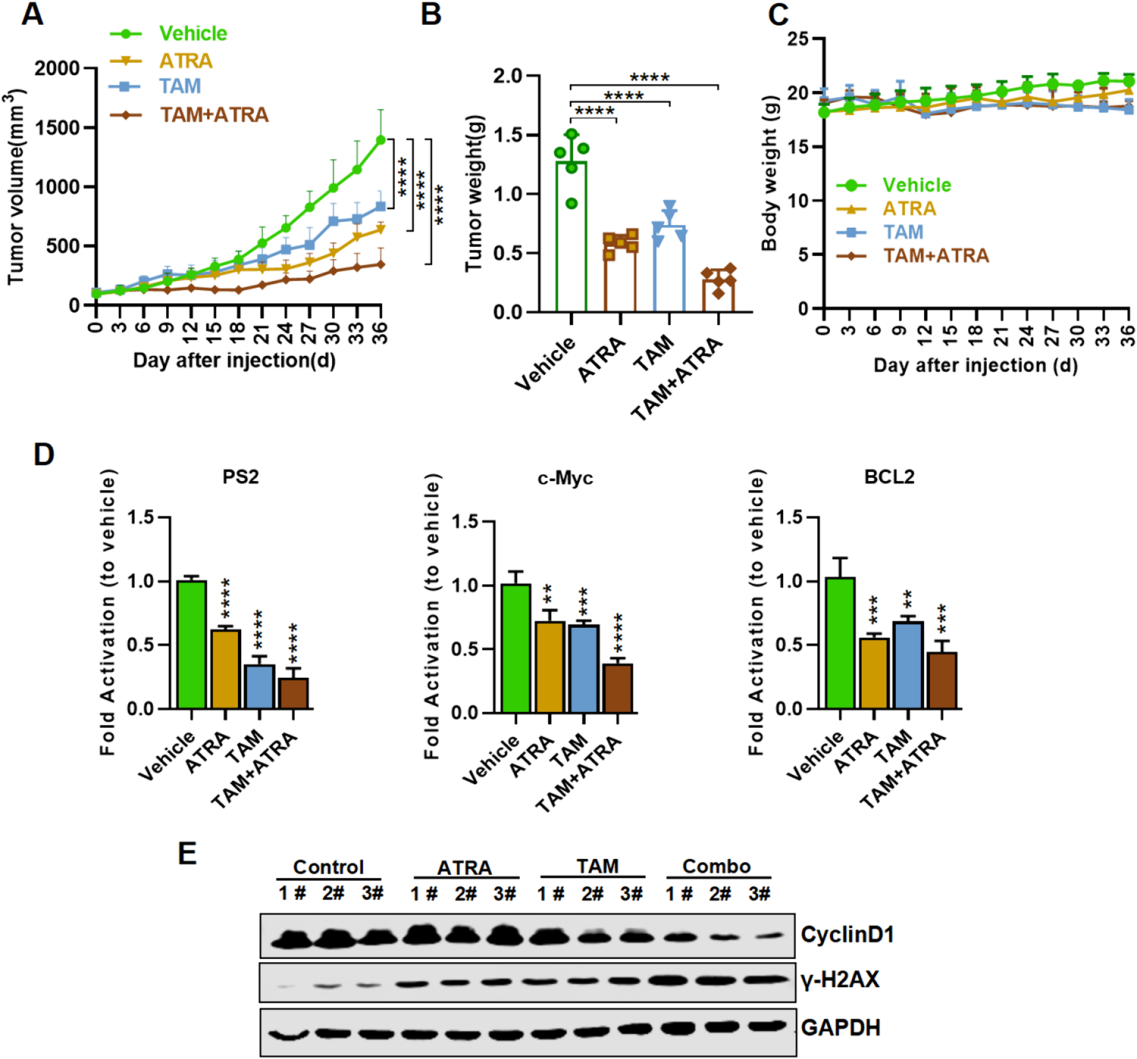
ATRA combined with TAM suppresses SKOV3-derived xenograft tumors in vivo. Female nude mice bearing SKOV3-derived tumors were randomized into four treatment groups: DMSO, ATRA (10 mg/kg, daily by i.p.), TAM (20 mg/kg, daily by i.p.), and ATRA+TAM. After 36 days of treatment, tumor volumes were measured every 3 days(**A**). The tumors were weighed (**B**) and body weight change was measured (C). PS2, c-Myc and BCL2 mRNA expression in each group of tumour samples by QPCR **(D)**. Three tumor samples were selected from each of the four groups, and the expressions of CyclinD1 andγ-H2AX were detected by western blot (**E**)

Based on the cellular level data, we established an ovarian cancer xenograft model in vivo by subcutaneously injecting SKOV3 cells into nude mice. Figure 6 A and Fig. 6 E show that ATRA combined with Tamoxifen suppressed SKOV3-derived xenograft tumor growth in vivo. Female nude mice bearing SKOV3-derived tumors were randomized into four treatment groups: DMSO, ATRA, Tamoxifen, and ATRA plus Tamoxifen. Figure 6A shows the change in tumor volume after DMSO, ATRA, Tamoxifen, and ATRA plus Tamoxifen treatment for 36 days. Compared to the DMSO control, both ATRA and Tamoxifen inhibited tumor growth approximately fourfold after 36 days of the injection, the combination of ATRA and Tamoxifen dramatically inhibited tumor growth compared to the other groups. The change in tumor weight exhibited a similar trend (Fig. 6B), but the body weight of the mice after the injection of different compounds was very similar across the groups, indicating that ATRA, Tamoxifen, and ATRA plus Tamoxifen have negligible toxicity (Fig. 6C). QPCR was carried out with RNA obtained from tumor tissues, and it was found that the expression of ER-related genes PS2, c-Myc and BCL-2 in tumor tissues was significantly inhibited in the experimental groups treated with ATRA combined with Tamoxifen (Fig. 6D). Western blot assay showed that the change of CyclinD1 and γH2AX expression. (Fig. 6E). These in vivo data demonstrate that ATRA in combination with Tamoxifen is an effective anti-cancer treatment strategy with minimal to no toxicity.

## Discussion

With advances in cancer diagnostics and precision medicine, clinical trials and ongoing research are continually evaluating new drugs and treatment options for ovarian cancer using combination therapies. In spite of this, ovarian cancer is difficult to diagnose and there are no targeted treatments, its morbidity and mortality are still at the forefront of malignant tumors. The database cases show that a positive correlation exists between the expression of estrogen receptor ERα and poor prognosis in ovarian cancer, and the expression of ERα in ovarian tumors is also higher than that in paracancerous tissue samples, which shows that ER is an important transcription factor that promotes the occurrence and development of ovarian cancer [20]. Tamoxifen inhibits the transcriptional function of ERα by antagonizing ER, thereby inhibiting the proliferation of ER-positive tumors, etc., and tamoxifen resistance is a complex and multifactorial phenomenon, one of which is the estrogen receptor (ER) Acquired Mutations: Mutations in the ERα gene result in structural changes to the receptor, making it less responsive to tamoxifen binding [21]. This reduces the drug's ability to block estrogen signaling and allows cancer cells to continue growing [22]. Through our research, we found that the combination of ATRA and TAM can inhibit the growth of ERα-positive ovarian cancer. We explored its mechanism and found that the combination can inhibit the downstream genes related to the transcriptional function of ERα, including GREB1, PGR and PS2, which are all involved the estrogen signaling pathway, is highly expressed in estrogen receptor-positive breast cancer cells, and its expression is often upregulated by estrogen stimulation. Moreover, ATRA combined with TAM can promote the apoptosis and cycle arrest of ER-positive ovarian cancer by affecting the genes related to cell cycle and apoptosis, and model tumors bearing subcutaneous tumors are inhibited in the proliferation of tumor cells. Our study solved the limitations of TAM monotherapy, which may lead to recurrence and drug resistance, and also verified that ATRA can regulate ERα-related signaling pathways in ERα-positive ovarian cancer, explaining the mechanism of the combination of the two drugs.

There are many studies on ATRA in hematological tumors, especially in the treatment of APL. However, due to the short half-life of ATRA, its role in solid tumors is limited, so there are relatively few studies on the treatment of solid tumors. Based on current research reports, ATRA also has good in vivo and in vitro effects in different tumors. In lung cancer, ATRA pretreatment can resist cisplatin resistance and can inhibit the proliferation of CD133 + cells induced by cisplatin resistance, suggesting that ATRA May inhibit genes related to stem cells [23]. In nasopharyngeal carcinoma, low concentration of ATRA combined with cisplatin can promote cancer death [24]. More studies have reported some mechanisms of ATRA promoting cancer cell death, for example, ATRA induces autophagic flux in breast cancer through RARα activation, in addition, using different RAR agonists and RARα knockdown breast cancer cells, proved that autophagy phagocytosis is dependent on RARα activation. ATRA treatment markedly increased apoptosis and attenuated epithelial differentiation. This points to a potential novel therapeutic strategy for the group of breast cancer patients applying both ATRA and autophagy inhibitors [25]. Therefore, the mechanisms by which ATRA works in different tumors are different, and exploring the potential combination of ATRA may provide different ways for tumor treatment. We will also further explore the deeper mechanism by which ATRA and TAM can synergistically treat ERα-positive ovarian cancer, which can provide some new ideas and strategies for the clinical treatment of ovarian cancer combined drugs.

## Data Availability

Data will be made available on request.
